# Carbonic anhydrase IX and acid transport in cancer

**DOI:** 10.1038/s41416-019-0642-z

**Published:** 2019-12-10

**Authors:** Holger M. Becker

**Affiliations:** Institute of Physiological Chemistry, University of Veterinary Medicine Hannover, D-30559 Hannover, Germany

**Keywords:** Cancer, Cell biology

## Abstract

Alterations in tumour metabolism and acid/base regulation result in the formation of a hostile environment, which fosters tumour growth and metastasis. Acid/base homoeostasis in cancer cells is governed by the concerted interplay between carbonic anhydrases (CAs) and various transport proteins, which either mediate proton extrusion or the shuttling of acid/base equivalents, such as bicarbonate and lactate, across the cell membrane. Accumulating evidence suggests that some of these transporters interact both directly and functionally with CAIX to form a protein complex coined the ‘transport metabolon’. Transport metabolons formed between bicarbonate transporters and CAIX require CA catalytic activity and have a function in cancer cell migration and invasion. Another type of transport metabolon is formed by CAIX and monocarboxylate transporters. In this complex, CAIX functions as a proton antenna for the transporter, which drives the export of lactate and protons from the cell. Since CAIX is almost exclusively expressed in cancer cells, these transport metabolons might serve as promising targets to interfere with tumour pH regulation and energy metabolism. This review provides an overview of the current state of research on the function of CAIX in tumour acid/base transport and discusses how CAIX transport metabolons could be exploited in modern cancer therapy.

## Background

Solid tumours are highly active tissues that often contain hypoxic regions, and produce vast amounts of metabolic acids. Through the constant release of acid, tumour cells create a hostile environment that fosters tumour growth and simultaneously kills adjacent host cells. Increased production and release of protons, in combination with restricted perfusion and alterations in the pH regulatory mechanisms lead to severe alterations in intracellular and extracellular pH, with significant consequences for tumour development and progression.^1–4^ While extracellular pH (pH_e_) can decrease to values as low as pH 6.5,^5–7^ intracellular pH (pH_i_) becomes slightly alkaline in cancer cells.^3,8^ This reversal in the pH gradient has been shown to occur at an early point in malignant transformation and can further increase with proceeding tumour growth.^9,10^ Extracellular acidification promotes tumour progression via various mechanisms, including pH-dependent modulation of integrin-mediated cell–matrix adhesion, degradation of the extracellular matrix via activation of cathepsins and various matrix metalloproteases and by killing of adjacent host cells.^4,11,12^ Furthermore, an acidic pH_e_ has been shown to suppress immunity, for example, by inhibition of chemotaxis or blocking of T-cell activation.^13,14^ Alkaline pH_i_, on the other hand, fosters cell proliferation^15–19^ and can limit apoptosis by suppression of caspase activity or alteration of mitochondria-dependent apoptosis.^20–22^ Furthermore, alkaline pH_i_ supports cancer cell migration by reorganisation of the cytoskeleton via pH-dependent activation of cofilin and talin, as well as membrane blebbing, which facilitates invasion and metastasis.^11,23,24^ Finally, alkaline pH_i_ triggers glycolytic activity, possibly by pH-mediated alterations in the activity of different glycolytic enzymes, which in turn results in increased acid production and exacerbates extracellular acidification.^25–27^ Taken together, these alterations in metabolism and pH regulation provide an evolutionary advantage for cancer cells over their surrounding host cells, and thereby contribute to somatic evolution, which selects for more aggressive phenotypes of cancer entities.^1,28,29^ Nevertheless, even though metabolic alterations and reversed pH gradient can pose an obstacle for conventional cancer therapies, they might also become a tumour’s Achilles’ heel to be exploited for novel therapeutic approaches.^3,14,30–34^

## Acid/base regulation in tumour cells

**Fig. 1:**
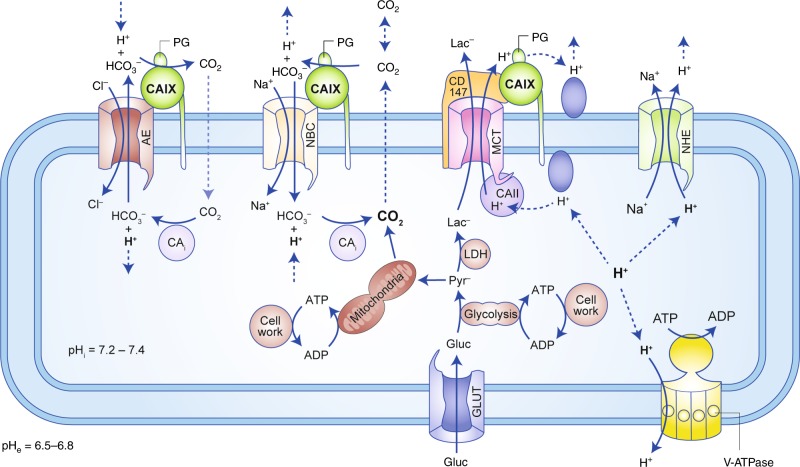
Tumour pH is regulated by the concerted interplay between acid/base transporters and carbonic anhydrase. Metabolic acids are produced by glycolysis and mitochondrial respiration. Anaerobic glycolysis yields lactate and H^+^ that are excreted from the cell by monocarboxylate transporters (MCTs) in a 1:1 stoichiometry. Mitochondrial respiration produces CO_2_, which is hydrated in the cell, forming HCO_3_^−^ and H^+^. CO_2_ can leave the cell by passive diffusion over the plasma membrane or through gas channels (not shown). Efficient pH regulation requires the function of additional transporters and enzymes, which either export protons from the cell or mediate the reimport of HCO_3_^−^. Additional export of H^+^ can be mediated by the Na^+^/H^+^ exchanger 1 (NHE) and by vacuolar H^+^-ATPase (V-ATPase). CO_2_ venting is further supported by the catalytic function of the extracellular carbonic anhydrase (CA) isoforms CAIX and CAXII (the latter one is omitted from this cartoon for clarity). Extracellular CAs catalyse the hydration of CO_2_ to HCO_3_^−^ and H^+^ at the membrane. HCO_3_^−^ can diffuse away from the cell or can be reimported by Na^+^/HCO_3_^–^ cotransporters (NBC) to support intracellular buffering. The extracellular HCO_3_^−^ can either be formed from ‘endogenous' CO_2_, which is produced by the cell through mitochondrial respiration or titration of HCO_3_^−^ and H^+^, or from extracellular CO_2_, produced from distant sources. Cl^−^/HCO_3_^−^ exchangers (AEs) have been suggested to function either as HCO_3_^−^ importers for pH buffering or HCO_3_^−^ exporters that extrude HCO_3_^−^ to load cellular compartments with Cl^–^ during cell migration. Transport activity of many acid/base transporters is facilitated by interaction with intracellular and extracellular CAs. NBC and AE interact with CAII and CAIX that either provide or remove HCO_3_^−^ to/from the transporter via their catalytic function. MCTs form a protein complex with CAII and CAIX, in which the CAs function as ‘proton antenna’ for the transporter, which mediates the rapid exchange of H^+^ between transporter pore and the surrounding protonatable residues.

Tumour pH regulation requires the concerted interplay between various acid/base transporters and carbonic anhydrases (CAs), as summarised  in Fig. 1. Cancer cells meet their demand for energy mainly by aerobic respiration of glucose or anaerobic glycolysis. Both pathways result in the formation of metabolic acids—either in the form of CO_2_, which hydrates to HCO_3_^–^ and H^+^, or in the form of lactate^–^  (Lac^–^) and H^+^ (Fig. 1). CO_2_ and Lac^–^/H^+^ have to be removed from the cell to avoid intracellular acidosis. CO_2_ can leave the cell by passive diffusion through the plasma membrane or via gas channels, such as aquaporins.^35,36^ Lactate, as a charged ion, cannot diffuse over the cell membrane, but is removed from the cell via monocarboxylate transporters (MCTs) in cotransport with H^+^ in a 1:1 stoichiometry^37–40^ (Fig. 1). Lactate transport in cancer cells is primarily mediated by the two isoforms MCT1 (SLC16A1) and MCT4 (SLC16A3), the expression of which has been shown to be upregulated in various tumour types, including breast cancer, gliomas, colorectal carcinomas and prostate cancer.^41–46^ An alternative pathway for lactate removal is passive diffusion of the ion through gap junctions, formed by connexins between adjacent tumour cells.^47^ Lactate is thereby dissipated from glycolytic cancer cells to recipient cells, situated in better perfused regions of the tumour, from where it can either be released to the pericellular space or serves as fuel for oxidative energy production.^47^ However, removal of metabolic acid by passive diffusion, either through cells or over the plasma membrane alone, is not sufficient for efficient cellular pH regulation, since restricted perfusion in the intracellular and extracellular space will lead to accumulation of the acids in the cell.^48,49^ Furthermore, sole removal of metabolic acids via MCTs and CO_2_ diffusion would render cytosolic pH dependent on the cell’s metabolic rate.^49^ Therefore, additional pH regulatory proteins are required to govern cellular pH. One of the major proton extruders in mammalian cells is the Na^+^/H^+^ exchanger NHE1 (SLC9A1). NHE1 is already upregulated in cancer cells at an early stage in tumour development,^9^ and the oncogene-dependent activation of NHE1, which results in cytosolic alkalinisation and extracellular acidification, has been suggested as a key mechanism in malignant transformation and tumour progression.^3,9,50^ Accumulating at the leading edge of lamellipodia,^51^ NHE1 promotes tumour cell migration and invasion of cancer cells by generating a pH gradient along the cell, with an acidic pH_e_ and alkaline pH_i_ at the migrating front.^52–55^ In addition, NHE1-mediated proton export at the protruding front supports digestion of the extracellular matrix by local acidification.^56,57^

Protons are also removed from the cell against their electrochemical gradient by vacuolar H^+^-ATPases, which are targeted to the plasma membrane of various cancer cells, including glioma, pancreatic cancer, hepatocellular carcinoma and oral squamous cell carcinoma, where the pumps have been shown to promote cell proliferation, migration and invasion.^58–67^

Intracellular pH is not only maintained by constant extrusion of protons, but also by import of HCO_3_^−^ via Na^+^/HCO_3_^−^ cotransporters such as NBCe1 (SLC4A4) and NBCn1 (SLC4A7). NBCs were shown to be the predominant net acid extruders in breast cancer tissue, where they facilitate tumour cell proliferation by counteracting metabolic acidosis.^50,68–71^ Furthermore, NBCs have been demonstrated to play a role in cell migration—NBCe1 is redirected to the leading edge,^72^ where NBC-mediated import of HCO_3_^−^ (together with NHE1-mediated H^+^ extrusion) leads to intracellular alkalinisation, in turn driving cytoskeletal remodelling.^11,72^ Cancer cell migration is further supported by the functional interaction between NBCe1 and the Cl^−^/HCO_3_^−^ exchanger AE2 (SLC4A2).^11,72^ It was suggested that AE2 imports osmotically active Cl^–^ in exchange for HCO_3_^−^, to support osmotic cell swelling.^11,72^ AE2 and NBCe1 would thereby work together to produce an ‘HCO_3_^−^ short circuit’, driving Cl^−^ influx into the cell during cell migration. For an in-depth discussion of pH regulation in cancer cells see also refs. ^3,4,11,48,49,73–76^ In healthy cells, activity of acid/base transporters is tightly regulated in an allosteric way. Transport activity of AE2 increases with alkaline pH_i/e_ and decreases with acidic pH_i/e_.^77–79^ Allosteric regulation of AE2 transport activity involves independent H^+^ sensing of the transporter by amino acid clusters in the cytosolic N-terminal domain and a small region in the transmembrane domain.^77–81^ Transport activity of NHE1, on the other hand, is allosterically activated by high intracellular proton concentrations and becomes quiescent at alkaline pH.^82–84^ Activation and inactivation of the transporters ensures tight control of pH_i_ and pH_e_. In cancer cells, however, these regulatory processes can be disturbed, contributing to dysregulation of pH_i_ and pH_e_. In cancer cells, transport activity of NHE1 was found to be activated by growth factors, hypoxia, acidic pH_e_, low serum concentration or by activation of CD44 by hyaluronan.^9,85–88^ For a detailed discussion on the regulation of acid/base transporters see refs. ^10,18,79,89^

## The role of CAIX in tumour acid/base regulation

Besides the concerted interplay between various acid/base transporters, effective acid venting and pH regulation requires the catalytic activity of intracellular and extracellular CAs, to catalyse the reversible hydration of CO_2_ to HCO_3_^−^ and H^+^.

Out of the six evolutionary distinct classes of CAs (α, β, γ, δ, ζ and η), only the α-class is expressed in mammals.^90^ The α-class of CAs comprises 16 isoforms, which vary regarding their catalytic activity and subcellular localisation. From the 12 catalytically active isoforms, expressed in humans, five are localised in the cytosol (CAI, CAII, CAIII, CAVII and CAXIII) and four are tethered to the plasma membrane with their catalytic domain facing the extracellular space (CAIV, CAIX, CAXII and CAXIV).^91–93^ CAVA and CAVB are expressed in the mitochondrial matrix while CAVI is secreted to the saliva.^91,92^ Three isoforms display no catalytic CA activity (CAVIII, CAX and CAXI). Therefore, these three proteins, which are mainly expressed in the central nervous system, are also termed CA-related proteins (CARPs).^94,95^

Cancer cells primarily express the plasma-membrane-associated CA isoforms CAIX and CAXII, as well as intracellular CAs such as CAI and CAII.^96–104^ Amongst the cancer-related CAs, CAIX has gained most attention, since expression of this isoform in healthy tissue is restricted to epithelial cells in the stomach and gut, but is strongly upregulated in many tumour tissues.^105–107^ The CAIX protein comprises an extracellular-facing catalytic domain tethered to the plasma membrane with a single transmembrane domain, and a short intracellular C-terminal tail. In addition, CAIX features an N-terminal proteoglycan-like domain (PG domain), which is unique to CAIX within the CA family.^108–110^ The PG domain was shown to contribute to the assembly of focal adhesion contacts during cell migration^111,112^ and was suggested to function as a proton buffer to support CAIX catalytic activity.^113^ Furthermore, it might serve as proton antenna for monocarboxylate transporters to facilitate proton-coupled lactate flux.^114^

CAIX, the expression of which is under control of the hypoxia-inducible factor 1 (HIF-1), is predominantly located in chronically hypoxic tumour regions.^110,115^ However, CAIX can also be found in mild hypoxic or even normoxic regions, since the expression of CAIX can be activated by components of the mitogen-activated protein kinase (MAPK) pathway.^116,117^ CAIX is expressed in a wide range of tumour entities, including breast and colorectal cancer, glioblastoma, lung cancer and cervical squamous cell carcinomas, and is usually linked to poor prognosis.^98,118–123^ In line with the correlation between CAIX expression and poor prognosis, various studies have demonstrated that CAIX can protect tumour cells from intracellular acidosis and functions as a pro-migratory factor that drives migration and invasion by both catalytic and non-catalytic mechanisms, which fosters the formation of metastasis.^72,112,123–130^

CAIX catalyses the reversible hydration of CO_2_ at the exofacial site of the plasma membrane. This simple reaction puts CAIX in a central position for the regulation of pH_i_ and pH_e_.^127,131,132^ By conversion of cell-derived CO_2_ into HCO_3_^−^ and H^+^, CAIX can maintain a steep outward-directed CO_2_ gradient over the plasma membrane, thereby facilitating CO_2_ excretion, which results in extracellular acidification and a more alkaline pH_i_^131,132^ (Fig. 1). Furthermore, hydration of CO_2_ at the extracellular site of the plasma membrane allows the parallel diffusion of CO_2_, HCO_3_^–^ and H^+^ through the periplasm.^131,132^ This immediate removal of CO_2_ at the outer site of the plasma membrane is of particular importance for poorly perfused but metabolically active tissue, such as solid tumours, where diffusion distances to the next blood capillary pose a barrier for the diffusive removal of metabolic waste products.^131,132^ Some HCO_3_^−^, produced by CAIX at the cell membrane, is reimported into the cell by adjacent HCO_3_^–^ transporters, like NBCe1 or NBCn1 (Fig. 1). In the cytosol, HCO_3_^−^ titrates H^+^ to generate membrane-permeable CO_2_, which diffuses out of the cell and contributes to net H^+^ extrusion. Efficient reimport of CAIX-derived HCO_3_^–^ requires a close proximity of CAIX to the bicarbonate transporters (Fig. 1). This close proximity could be achieved by direct interaction between the proteins, by forming a complex coined the ‘transport metabolon’, as described in the next section.

Even though an acidic microenvironment fosters tumour progression, pH_e_ must not drop too low in order to avoid over-acidification and necrosis of cancer cells. It was therefore suggested that CAIX can set the pH of the tumour microenvironment to a moderate acidic value, which provides an advantage to cancer cells, while preventing over-acidification-induced cell death.^90,131–136^ In contrast to other CA isoforms, CAIX is most active at pH 6.8, a value that closely resembles tumour pH_e_.^133,134^ At pH values above 6.8, the rate of the hydration reaction (H^+^ production) is higher than the rate of dehydration, while at pH values below 6.8 the rate of the dehydration reaction (H^+^ consumption) exceeds the rate of the hydration reaction.^133,134^ This implies that at a pH_e_ above 6.8, CAIX produces H^+^ by the hydration of CO_2_, to acidify the pericellular space. At a pH_e_ below 6.8, CAIX removes H^+^ by the dehydration reaction, to counteract further acidification. In line with this hypothesis, Lee et al.^135^ recently showed that CAIX not only contributes to acidification of the extracellular space, but also functions as a ‘pH-stat’ to stabilise pH_e_ at a moderate acidic value in spheroids and tumour xenografts. Since this moderate acidity is well tolerated by cancer cells but could be lethal to normal cells, setting pH_e_ to a constant acidic condition might pose an evolutionary strategy in cancer cells—this creates an environment that fosters tumour growth and invasion in response to microenvironmental selection forces.^70,137^

The various roles of CAIX in tumour acid/base regulation have been extensively discussed in a variety of excellent reviews.^73,90,110,130,138,139^ Therefore, the review will not further deepen the discussion of the general functions of CAIX in tumour pH regulation but focus on the role of CAIX in the facilitation of acid/base transporters in tumour cells.

## Transport metabolons

**Fig. 2:**
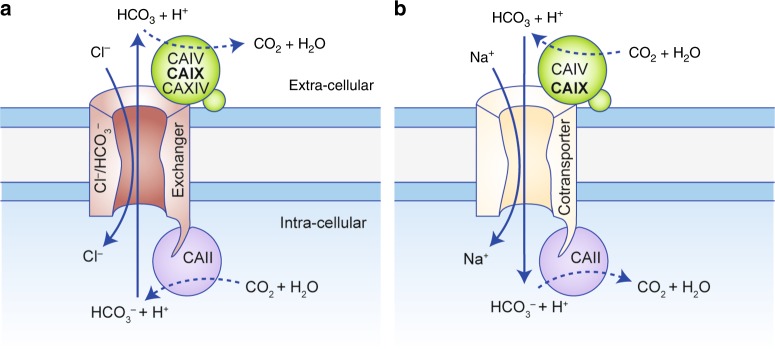
Bicarbonate transport metabolons with carbonic anhydrase. (**a)** Cl^–^/HCO_3_^−^ exchangers (AEs) and (**b**) Na^+^/HCO_3_^−^ cotransporters (NBCs) form *bicarbonate transport metabolons* with intracellular and extracellular carbonic anhydrases (CAs). Cytosolic CAII binds to the transporter’s C-terminal tail. Extracellular CAs, which are tethered to the plasma membrane by a transmembrane domain (CAIX, CAXIV) or GPI anchor (CAIV), bind to the transporter’s fourth extracellular loop. By catalysing the reversible hydration of CO_2_ to HCO_3_^−^ and H^+^ in the immediate vicinity of the transporter, intracellular and extracellular CAs either provide or remove HCO_3_^−^ to/from the transporter. Through this mechanism, they suppress the depletion of HCO_3_^−^ at the *cis*-side of the transporter and HCO_3_^−^ accumulation at the *trans*-side, which, in turn, drives HCO_3_^−^ flux across the cell membrane.

Various acid/base transporters have been shown to both physically and functionally interact with intracellular and extracellular CAs to form a protein complex coined the ‘transport metabolon’. A metabolon has been defined as a ‘temporary, structural-functional, supramolecular complex of sequential metabolic enzymes and cellular structural elements, in which metabolites are passed from one active site to another, without complete equilibration with the bulk cellular fluids (channelling)’.^140–142^ The most extensively studied CA isoform to interact with acid/base transporters is the intracellular CAII. CAII binds to an acidic cluster in the C-terminal tail of various HCO_3_^−^ transporters, including the Cl^–^/HCO_3_^–^ exchangers AE1, AE2 and AE3,^143–147^ as well as the Na^+^/HCO_3_^–^ cotransporter NBCe1^148,149^ and NBCn1 (NBC3).^150,151^ Direct binding of CAII to the transporter’s C-terminal tail was suggested to maximise the local HCO_3_^−^ concentration in the direct vicinity of the transporter pore.^147^ Indeed, inhibition of CAII catalytic activity by sulfonamides such as acetazolamide or ethoxyzolamide, as well as co-expression of AEs and NBCs with the catalytically inactive CAII mutant CAII-V143Y, resulted in a loss of the CAII-mediated increase in transport function.^147,151–153^ Taken together, it can be assumed that CAII, directly bound to the C-terminal tail of AEs and NBCs, provides (or removes) HCO_3_^−^ to (from) the transporter by the reversible hydration of CO_2_ (Fig. 2a, b). It was suggested that CAII would maximise HCO_3_^−^ flux by maximising the transmembrane HCO_3_^−^ gradients in the immediate vicinity of the transporter.^147,154^ AE1, for example, has a turnover rate of 5 × 10^4^ s^−1^ and would therefore rapidly deplete the *local* substrate pool. The transport metabolon between CAII and AE could increase HCO_3_^−^ transport by minimising the distance of substrate diffusion between enzyme and transporter, thereby increasing substrate availability for the transporter.^147,154^

HCO_3_^−^ transporters do not only interact with intracellular CAII, but also with extracellular isoforms, such as CAIV, CAIX and CAXIV^155–157^ (Fig. 2a, b). A physical and functional interaction between HCO_3_^−^ transporters and CAIX was demonstrated in 2007 by Patricio Morgan et al. in vitro and in HEK293 cells.^157^ By applying co-immunoprecipitation experiments with AEs and CAIX in HEK293 cells and pull-down assays with GST-fusion proteins, the authors could demonstrate that the catalytic domain of CAIX directly binds to AE1, AE2 and AE3. The authors could further show that CAIX increases transport activity of AE1–3 when the proteins were co-expressed in HEK293 cells.^157^ Interestingly, CAIX did not alter transport activity of the putative Cl^–^/HCO_3_^−^ exchanger SLC26A7. Furthermore, SLC26A7 could not be co-precipitated with CAIX, indicating no physical interaction between the two proteins. These results suggest that the direct interaction between CAIX and AEs is indeed a prerequisite for the CAIX-mediated facilitation of AE transport activity.

CAs can also from transport metabolons with NHEs. CAII was shown to bind to and enhance transport activity of NHE1 and NHE3.^158–161^ Furthermore, it was suggested that extracellular CAIV can facilitate NHE transport activity in rabbit non-pigmented ciliary epithelium cells.^162^ In both cases, facilitation of NHE transport function required CA catalytic activity. These results suggest that CAII and CAIV facilitate NHE transport activity by a similar mechanism as demonstrated for bicarbonate transporters. First evidence that CAIX might form a transport metabolon with NHE1 in cancer cells was recently provided by Liskova et al.^163^ The authors could co-precipitate NHE1 with CAIX and the Na^+^/Ca^2+^ exchanger NCX1 from lysates of hypoxic SiHa cells. From these and other data, the authors suggested that the three proteins, which are colocalised in hypoxic SiHa cells (as shown by an in situ proximity ligation assay), can form a transport metabolon that contributes to tumour pH regulation.^163^

Despite the constantly increasing number of publications in favour of the concept of the transport metabolon, the existence of these protein complexes has also been questioned by several studies.^164–167^ One major point of criticism relates to the direct interaction between transporter and enzyme. Most binding studies were performed by using GST-fusion proteins of the transporters’ C-terminal tails,^147,150,151,155,158,159^ which were criticised to result in false-positive binding.^165^ Piermarini et al.^165^ could reproduce previous findings that CAII binds to a GST-fusion protein of the C-terminal tails of AE1, NBCe1 and NDCBE. However, when performing the assay with pure peptides, no binding to CAII could be observed.^165^ The concept of a bicarbonate transport metabolon was also fundamentally questioned by a study from Al-Samir et al.^167^, who examined the interaction between AE1 and CAII in native human red blood cells and the tsA201 human embryonic kidney cell line. The authors did not find convincing evidence for a direct interaction between AE1 and CAII. Furthermore, their mathematical models favoured equal distribution of CAII throughout the cytosol over accumulation of the enzyme at the cell membrane for efficient HCO_3_^–^ transport.^167^ Based on these results, it was suggested that CAs could improve bicarbonate supply to membrane transporters without the necessity to form a physical protein complex.^167^ An in-depth discussion of the various types of interactions between acid/base transporters and CAs, including the controversies on transport metabolons, is provided in a number of recommendable reviews.^142,154,168,169^

## Role of bicarbonate transport metabolons with CAIX in cancer cell motility

Transport metabolons have been suggested to play a role in different physiological processes, including gas exchange in erythrocytes,^144^ acid/base regulation in the heart,^170,171^ brain and retina,^160,172,173^ gastric acid secretion^157,174^ and the reabsorption of salt and water in kidney and intestine.^161^

First evidence for a bicarbonate transport metabolon in cancer cells was provided by the laboratory of Silvia Pastorekova and Jaromir Pastorek in hypoxic squamous cell carcinoma and lung carcinoma cells.^72,175^ The authors demonstrated that CAIX accumulates in the lamellipodia of hypoxic A549 lung carcinoma cells, where it colocalised with the Na^+^/HCO_3_^−^ cotransporter NBCe1.^72,175^ CAIX also colocalised with the Cl^–^/HCO_3_^–^ exchanger AE2 in the leading edge of SiHa squamous cell carcinoma cells, which migrated from hypoxic spheroids. An in situ proximity ligation assay, which detects colocalisation of proteins with a maximum distance of 40 nm between the epitopes in native cells, suggested that CAIX directly interacts with NBCe1 and AE2 in the lamellipodia of migrating A549 and SiHa cells.^72^

Both NBCe1 and AE2 have been attributed a central function for cell migration. NBCe1 contributes to the reversal of the pH gradient, which is required for intracellular remodelling of the actin cytoskeleton and extracellular detachment from the matrix. By catalysing the hydration of CO_2_ at the cell membrane, CAIX ensures local availability of extracellular HCO_3_^−^ for direct import via the NBCe1 to increase intracellular buffer capacity. The remaining protons contribute to the acidification of the pericellular space and drive tumour cell invasiveness.^175^ The exact role of AE2 in cell migration, however, is still under debate. AE2 might extrude HCO_3_^−^ in exchange to osmotically active Cl^–^ to support cell swelling.^11,72^ CAIX, which directly interacts with NBCe1 and AE2 at the leading edge of migrating cancer cells, could provide (or remove) HCO_3_^−^ to (from) the transporters to support their transport activity. Indeed, inhibition of CAIX activity—either by application of acetazolamide or overexpression of a CAIX mutant—lacking the catalytic domain, resulted in a significant reduction of migratory activity in hypoxic HeLa cells.^72^ Based on these results, it appears plausible that the transport metabolons, formed between NBCe1, AE2 and CAIX in the lamellipodia of migrating cells, can contribute to the reversal of the pH gradient and Cl^−^-mediated swelling at the protruding front of the cell to drive cell migration.

## Non-catalytic transport metabolons with CAIX and MCTs

**Fig. 3:**
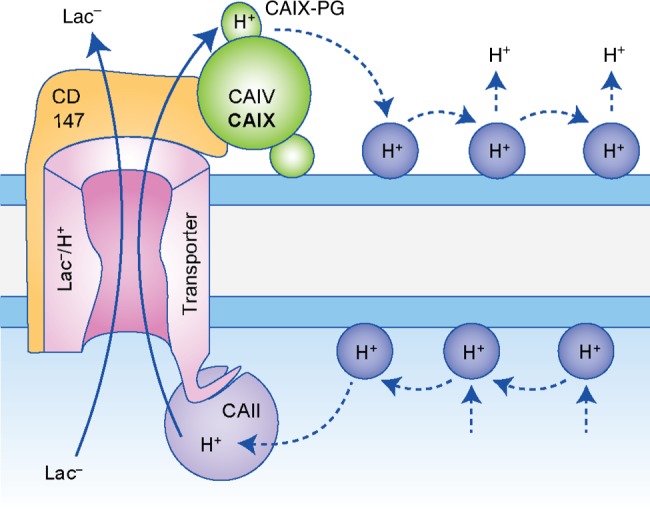
Carbonic anhydrases function as proton antenna for monocarboxylate transporters. The monocarboxylate transporter isoforms 1 and 4 (MCT1/4) form a non-catalytic transport metabolon with CAII, CAIV and CAIX. This type of interaction is independent from carbonic anhydrase (CA) catalytic activity. Intracellular CAII, which is bound to the MCT1/4 C-terminal tail, functions as proton antenna for the transporter, which mediates the rapid exchange of H^+^ between transporter pore and surrounding protonatable residues (blue–grey circles) at the inner face of the plasma membrane. On the extracellular site, CAIV and CAIX, which are bound to the Ig1 domain of the MCT chaperone CD147, mediate shuttling of protons between the transporter and protonatable residues at the extracellular face of the plasma membrane. In CAIX, proton shuttling is mediated by the CAIX–PG domain. The proton shuttle in CAIV is yet unidentified.

Tumour cells, especially those that reside in a hypoxic environment, display a substantial increase in glycolytic activity, resulting in increased production of lactate and protons. Lactate and protons are removed from the cell via the monocarboxylate transporters MCT1 and MCT4, which contributes to the formation of an acidic microenvironment. Lactate transport was found to be increased in hypoxic breast cancer cells.^176^ Knockdown of CAIX, however, abolished the hypoxia-induced increase in lactate flux.^176^ Surprisingly, inhibition of CAIX enzymatic activity with ethoxyzolamide had no effect on MCT transport activity, indicating that CAIX facilitates MCT transport activity by a mechanism that is independent from the enzyme’s catalytic function.^176^ In line with these results, Crispr-mediated knockout of CAIX decreases proton excretion rates from glycolysis (GlycoPER) in the triple-negative breast cancer cell line UFH-001.^136^ Isoform-specific inhibition of CAIX with three different ureido-substituted benzene sulfonamides (USBs), however, did not change GlycoPER, suggesting a non-catalytic function of CAIX in glycolysis-derived acid secretion.^136^ Such a non-catalytic facilitation of MCT transport activity was previously observed in *Xenopus laevis* oocytes, heterologously expressing MCT1/4 and intracellular CAII.^177–185^ CAII binds to an acidic cluster in the C-terminal tail of MCT1 (E^489^EE) and MCT4 (E^431^EE), but not to MCT2.^182,185^ Since CAII catalytic activity is not required to facilitate MCT1/4 transport activity, it was suggested that CAII could function as a ‘proton antenna’ for the transporter^180^ (Fig. 3). The physiological need for such a proton antenna results from the slow diffusion of highly buffered protons within the cytosol. Martinez et al.^186^ calculated that the maximum supply rate of H^+^ via diffusion through the cytosol is significantly lower than the apparent turnover rate of MCT1. In other words, MCTs extract H^+^ from their surrounding at a rate that exceeds the capacity for simple diffusion to supply H^+^ to the transporter. To solve this paradox, the authors suggested that the transporters extract H^+^ from surrounding ‘proton harvesting compartments’ and not directly from the cytosol.^186^ Like other CAs, CAII facilitates an intramolecular proton shuttle to move H^+^ between the catalytic centre and the surrounding bulk solution. Proton transfer is mediated by His^64^, which shuttles H^+^ between the bulk solvent and a well-ordered water wire in the enzyme’s active site cavity.^187^ Modelling studies further suggested that the active site proton pathway exits to the protein surface, leading to the two acid residues Glu^69^ and Asp^72^.^188^ Indeed, investigations in *Xenopus* oocytes showed that these two residues mediate proton transfer between MCT1/4 and CAII, while CAII–His^64^ is not involved in proton shuttling between enzyme and transporter but mediates binding of CAII to the MCT1/4 C-terminal tail.^184^ In analogy to the findings on CAII, it was suggested that CAIX could serve as an extracellular proton antenna for MCTs in cancer cells.^114,176,189^ The catalytic domain of CAIX seems to lack a homologue cluster to CAII–Glu^69^ and Asp^72^. However, the CAIX–PG domain features eight aspartate and 18 glutamate residues, which have been suggested to function as an intramolecular proton buffer for the enzyme.^113^ Indeed, co-expression of MCT1/4 with a truncation mutant of CAIX, missing the PG domain (CAIX–ΔPG), in *Xenopus* oocytes did not facilitate MCT transport activity.^114^ Furthermore, application of an antibody, mapping against the CAIX–PG domain, supressed CAIX-mediated facilitation of lactate transport in *Xenopus* oocytes and hypoxic breast cancer cells.^99^ Based on these results, it was suggested that the CAIX–PG domain functions as proton antenna, by mediating the rapid exchange of H^+^ between transporter and surrounding protonatable residues^114^ (Fig. 3). Interestingly, CAII and CAIX can facilitate MCT-mediated H^+^/lactate transport both in the inward and outward direction.^114,180^ During H^+^-coupled lactate efflux, as observed in lactate-producing cancer cells, intracellular CAII would collect H^+^ from surrounding protonatable residues near the inner face of the cell membrane and donate them to the transporter. On the extracellular site, CAIX would remove H^+^ from the transporter pore and transfer it to surrounding protonatable residues near the extracellular face of the plasma membrane (Fig. 3). During H^+^-coupled lactate influx (as found in lactate-consuming cell types) the shuttle would operate in the opposite direction.

While CAII directly binds to the transporter, physical interaction between MCT1/4 and CAIX is mediated by the transporters’ chaperone CD147.^189^ Pull-down assays demonstrated that CAIX binds to the Ig1 domain of CD147 by forming a hydrogen bond between CD147–Glu^73^ and CAIX–His^200^, the residue analogue to CAII–His^64^ and the central residue of the CAIX intramolecular proton shuttle.^189^ CAIX does not only interact with MCT1 and MCT4, but also with the high-affinity lactate transporter, MCT2,^189^ found in various tumour tissues, including breast carcinoma, colon adenocarcinoma, lung cancer, ovarian adenocarcinoma and prostate cancer.^190–192^ The chaperone of MCT2, GP70,^193^ features a positively charged lysine instead of a negatively charged glutamate at the CA-binding site.^189,194^ By combining pull-down experiments with electrophysiological studies in *Xenopus* oocytes, it was demonstrated that CAIV–His^88^ (the analogue residue to CAII–His^64^ and CAIX–His^200^) either serves as hydrogen donor or hydrogen acceptor, depending on the properties of its binding partner.^194^ Based on these findings, it was suggested that CAIX–His^200^ could also serve as hydrogen donor and acceptor to mediate binding of CAIX to different MCT chaperones.^189^ Binding of CAIX to the transporter’s chaperone is mandatory for the CAIX-mediated facilitation of MCT activity. This was shown by application of an antibody against the CD147–Ig1 domain that displaced CAIX from the transporter and decreased MCT-mediated lactate flux in *Xenopus* oocytes and breast cancer cells.^189^

The MCT1/4–CAIX transport metabolon was not only observed in cultivated breast cancer cells, but also in human tumour tissue. By use of an in situ proximity ligation assay (PLA), Ames et al. could recently demonstrate a direct interaction between MCT1, MCT4 and CAIX in tissue samples of human breast cancer patients.^189^ Interestingly, the number of PLA signals increased with higher tumour grade, indicating that the number of MCT1/4–CAIX transport metabolons increases during tumour progression.^189^

Not only is lactate transport in cancer cells facilitated by extracellular CAIX, but also by intracellular CAII.^184^ CAII was found to physically interact with MCT1 in MCF-7 breast cancer cells, as shown by PLA.^184^ Knockdown of CAII, but not inhibition of catalysis, decreased lactate transport in normoxic and hypoxic MCF-7 breast cancer cells and reduced cell proliferation.^176,184^ These results indicate that efficient lactate efflux from cancer cells requires both intracellular and extracellular CAs. That intracellular and extracellular CAs can work in concert to drive MCT-mediated lactate transport was demonstrated for CAII and CAIV in *Xenopus* oocytes.^181^ CAII and CAIV together increased MCT1 activity by a factor of up to 3.5, while each isoform alone increased MCT1 activity by a factor of 1.5–2.7.^181^ Since diffusion of H^+^ to/from the transporter pore is slow, H^+^-coupled lactate transport via MCTs would lead to a local depletion/accumulation of H^+^ in the immediate vicinity of the transporter pore (termed proton microdomain), which would impair MCT transport activity.^180,186^ A proton antenna on only one site of the membrane could not prevent formation of the proton microdomain on the other side. This proton microdomain prevents a further increase in transport function. Combination of an intracellular and an extracellular proton antenna would prevent formation of proton microdomains on both sides of the membrane and allow maximum transport activity. Therefore, it was suggested that intracellular and extracellular CAs co-operate by a ‘push and pull’ principle—pushing protons towards the transporter pore on one side of the membrane and pulling them away from the transporter on the other side^181^ (Fig. 3). In cancer cells, efficient efflux of lactate and protons would therefore require the concerted action of both extracellular CAIX and intracellular CAII. This assumption is supported by a mathematical model of proton-coupled lactate transport in cancer cells.^195^ The model suggested the existence of local H^+^ pools near the cell membrane, which influence MCT-mediated lactate transport. By functioning as proton antenna, CAII and CAIX control these proton pools to provide a stable proton gradient for the transporter and drive proton-coupled lactate flux across the membrane of hypoxic cancer cells.^195^

Taken together, these findings demonstrate that MCT1 and MCT4 form a transport metabolon with CAII and CAIX in cancer cells (Fig. 3). Intracellular CAII binds to an acidic cluster in the transporters’ C-terminal tail, while extracellular CAIX binds to the Ig1 domain of the transporters’ chaperone CD147. Binding brings the CAs close enough to the transporter to establish an efficient proton shuttle between transporter pore and surrounding protonatable residues at the cell membrane. This proton shuttling counteracts the formation of proton microdomains around the transport and drives the export of glycolysis-derived lactate and protons from the cancer cell (Fig. 1).

## Transport metabolons as potential therapeutic targets in cancer therapy

A variety of preclinical studies have demonstrated that inhibition of CAIX catalytic activity can decrease proliferation and metastatic potential of various types of tumour cells. The potential use of CAIX inhibitors for cancer therapy has been intensively discussed in various reviews^139,196–198^ and should therefore not be discussed again here. However, bicarbonate transport metabolons with CAIX, per se, have not been subject to preclinical investigations as drug targets in cancer cells. Nevertheless, since CAIX-mediated facilitation of bicarbonate transporters requires CAIX catalytic activity, it can be assumed that inhibition of CAIX activity by small-molecule inhibitors or antibodies also inhibits CAIX-mediated facilitation of acid/base flux via NBCs and AEs. Therefore, it appears plausible that the effects of CAIX inhibitors on tumour progression can be partly attributed to the interference with bicarbonate transport metabolons. Targeting acid/base transporters in cancer cells via CAIX might even be advantageous over direct inhibition of the acid/base transporters, since these proteins are usually expressed in a variety of healthy tissue, rendering cancer cell-specific targeting difficult. For example, the NHE1 inhibitor cariporide, which was a promising agent for treatment of myocardial infarction, failed in Phase 3 clinical trial due to severe side effects. These side effects have been attributed to the widespread expression of NHE1.^199,200^

Since CAIX-mediated facilitation of MCT transport activity is independent from the enzyme’s catalytic function, classical sulfonamide-based CAIX inhibitors will most likely not target the MCT–CAIX transport metabolon. Indeed, inhibition of total CA activity with ethoxyzolamide had no effect on lactate flux in hypoxic MCF-7 breast cancer cells.^176^ In line with that, a recent study demonstrated that inhibition of CAIX catalytic activity with ureido-substituted benzene sulfonamides, which selectively inhibit CAIX activity in breast cancer cells,^201^ does not suppress the CAIX-mediated facilitation of proton secretion in UFH-001 cancer cells.^136^ However, the application of antibodies directed against the CD147–Ig1 domain (anti-CD147) or the CAIX–PG domain (anti-PG) resulted in a significant decrease in lactate transport and reduced cell proliferation in hypoxic tumour cells.^114,189^ Anti-CD147 displaces CAIX from the transporter–chaperone complex, thereby acting as a ‘metabolon disruptor',^189^ while anti-PG was suggested to interfere with the shuttling function of CAIX.^114^ These findings provide a proof of concept that the MCT1/4–CD147–CAIX transport metabolon is a potential target that could be exploited to interfere with cancer cell metabolism to reduce cell proliferation and thereby inhibit tumour progression. Lactate transport in cancer cells could also be targeted by direct inhibition of MCTs.^202–204^ Indeed, the MCT1-specific inhibitor AZD3965 is currently in Phase 1 clinical trial (clinicaltrials.gov identifier: NCT01791595). Since MCT1 is ubiquitously expressed throughout the body, systemic MCT1 inhibition might lead to side effects. Therefore, inhibition of MCT1/4 transport activity by ‘disruption’ of the MCT1/4–CAIX transport metabolon might provide a more targeted approach than systemic blocking of the transporter via MCT1/4 inhibitors. However, without further investigations these thoughts remain purely speculative.

## Conclusion

Alterations in energy metabolism and acid/base homoeostasis are emerging hallmarks of cancer cells.^205,206^ Tumour pH regulation is governed by the concerted interplay between various acid/base transporters and CAs, some of which form a structural and functional complex, coined the ‘transport metabolon’. Transport metabolons with CAIX have been suggested to play fundamental roles in tumour metabolism and pH regulation. CAIX can directly interact with the HCO_3_^−^ transporters NBCe1 and AE2 in the leading edge of migrating cancer cells to facilitate HCO_3_^−^ flux across the plasma membrane and support the generation of a pH gradient at the cell’s protruding front, which drives cancer cell migration and thereby formation of metastasis.^72^ CAIX was further shown to function as a ‘proton antenna’ for the lactate transporters MCT1 and MCT4, which mediate the rapid exchange of H^+^ between transporter pore and surrounding protonatable residues to drive proton-coupled lactate transport across the cell membrane and allow tumour cells to keep up a high glycolytic rate under hypoxia.^114,176,189^ Based on these findings, transport metabolons might serve as promising targets to interfere with tumour pH regulation and energy metabolism. Even though numerous studies investigated the therapeutic benefit of CAIX inhibitors for cancer treatment,^196–198^ transport metabolons have not been studied as therapeutic targets until now. Indeed, it could be speculated that targeting acid/base transporters via their interaction with CAIX might even provide an advantage over a direct targeting, since these transporters are expressed in a wide range of tissue, posing the risk of severe side effects by direct inhibition. CAIX, however, is almost exclusively expressed in tumour cells. CAIX transport metabolons might therefore be more specific targets than the transporters themselves. Since CAIX-mediated facilitating of HCO_3_^−^ transport requires CAIX catalytic activity, conventional CAIX inhibitors can be expected to also inhibit bicarbonate transport metabolons in cancer cells. CAIX-mediated facilitation of proton-coupled lactate transport, however, appears independent from CAIX catalytic activity.^114,176,189^ New types of CAIX inhibitors have to be designed to target these transport metabolons, in order to suppress the direct interaction between enzyme and transporter, to interfere with lactate flux and thereby interfere with energy metabolism in tumour cells.

## Data Availability

Not applicable.
